# MicroRNAs expression in CD4 T cells from HTLV-1 individuals

**DOI:** 10.1186/1742-4690-11-S1-P111

**Published:** 2014-01-07

**Authors:** Kátia Kaori Otaguiri, Mariana Tomazini Pinto, Evandra Strazza Rodrigues, Virgínia Mara de Deus Wagatsuma, Maurício Cristiano Rocha Júnior, Osvaldo Massaiti Takayanagui, Dimas Tadeu Covas, Simone Kashima

**Affiliations:** 1 Regional Blood Center of Ribeirão Preto, University of São Paulo (USP), Brazil; 2 Faculty of Pharmaceutical Sciences of Ribeirão Preto, USP, Brazil; 3 Faculty of Medicine of Ribeirão Preto, USP, Brazil

## 

HAM/TSP is an inflammatory manifestation of central nervous system caused by HTLV-1 and the mechanism of HAM/TSP development is no well elucidated. Currently, a promising approach on the physiopathogenesis of viral infections has been the evaluation of microRNAs (miRNAs) role. There are few data involving CD4+ T cells miRNA expression in HTLV-1 infection as well as in HAM/TSP establishment. To identify miRNAs differentially expressed in CD4+ T cells among non-infected individuals (CT), asymptomatic (HAC) and HAM/TSP patients we applied quantitative real time PCR. The analysis of miRNA expression profile in these cells showed 56 and 10 miRNAs upregulated 1.5 times in HAM/TSP and HAC groups, respectively. miR-125b-1-1 was upregulated in HAC group and miR-146a in HAM/TSP. Target prediction by in silico analysis showed that IFNG was a potentially miR-125b-1-1 target and IRAK1 and TRAF6 were miR-146a targets. IFNG expression was 1.3 higher in HAC than CT group and 1.8 higher in HAM/TSP than CT group. It was observed that TRAF6 expression was 15.7 and 1.5 times higher in HAM/TSP and HAC groups, respectively. There was no difference of IRAK1 expression among the three groups. Overexpression assays of miR-125b-1-1 altered IFNG expression and overexpression of miR-146a altered IRAK1 gene and protein expression. The results revealed that miRNAs could modulate genes and proteins during HTLV-1 infection. miR-125b and IFNG gene correlation suggests that miR-125b seems to contribute to HAM/TSP development. Besides, interaction between miR-146a and IRAK1/TRAF6 suggests that miR-146a seems to contribute to HTLV-1 establishment in CD4+ T cells.

